# Solifluction rates and environmental controls at local and regional scales in central Austria

**DOI:** 10.1080/00291951.2017.1399164

**Published:** 2017-11-13

**Authors:** Andreas Kellerer-Pirklbauer

**Affiliations:** ^a^ Working Group Alpine Landscape Dynamics (ALADYN), Department of Geography and Regional Science, University of Graz, Heinrichstrasse 36, AT-8010 Graz, Austria

**Keywords:** Iva Svare Holand, Catriona Turner, freeze-thaw, gelifluction, needle-ice creep, permafrost, solifluction

## Abstract

Solifluction is a widespread periglacial phenomenon. Little is known about present solifluction rates in Austria. The author monitored five solifluction lobes during a four-year period. Annual rates of surface velocity, vertical velocity profiles, depths of movement, and volumetric velocities were quantified using near-surface markers and painted lines. Environmental conditions were assessed using air temperature, soil texture, and ground temperature-derived parameters. The latter were used to estimate the relevance of needle-ice creep, diurnal frost creep, annual frost creep, and gelifluction. The mean surface velocity rates were 3.5–6.1 cm yr^−1^ (near-surface markers) and 6.2–8.9 cm yr^−1^ (painted lines), respectively, indicating a high relevance of needle-ice creep. The mean depth of movement was 32.5–40 cm. The mean volumetric velocities were 71–102 cm^3^ cm^−1^ yr^−1^. Solifluction rates at the five sites did not correlate with each other due to site-specific controls. No statistically significant correlations were quantified between solifluction rates and different environmental parameters due to data gaps and short time series, thus highlighting the importance of long-term monitoring. Nevertheless, the results suggest that longer zero curtain periods, longer seasonal ground thawing periods, later start of the seasonal snow cover, more freeze-thaw cycles, and cooler early summer temperatures promote solifluction.

## Introduction

Solifluction is a widespread phenomenon in the alpine and subalpine ecotones of high mountain areas and in polar and subpolar regions (Matsuoka 2001). Solifluction is a distinctive type of periglacial mass wasting in both non-permafrost and permafrost settings (Harris 1987; Matsuoka 2001). The term solifluction was introduced more than 100 years ago by J.G. Anderson for general ‘soil flow’, in his classic paper on solifluction in the Falkland Islands and Bear Island (Anderson 1906). In modern scientific usage the term solifluction represents collectively slow mass wasting associated with freeze-thaw action including soil saturation occurring in periglacial environments (Ballantyne & Harris 1994; French 2007). Currently, solifluction is commonly classified as needle-ice creep, frost creep, gelifluction (the movement of saturated soil associated with ground thawing (Washburn 1979)), and plug-like flow (Matsuoka 2001). Landforms caused by solifluction reflect both the thickness of frost-susceptible soil and the depth subjected to freeze-thaw action (Matsuoka 2011). Rates and processes of solifluction depend on climate, hydrology, geology, sedimentology, topography, and vegetation (e.g. Jaesche et al. 2003; Matsuoka 2011). Understanding and predicting the evolution of periglacial landforms related to solifluction requires quantitative relationships between the rate of solifluction and these variables (Matsuoka 2001; 2011; Hjort 2014).

**Fig. 1.**
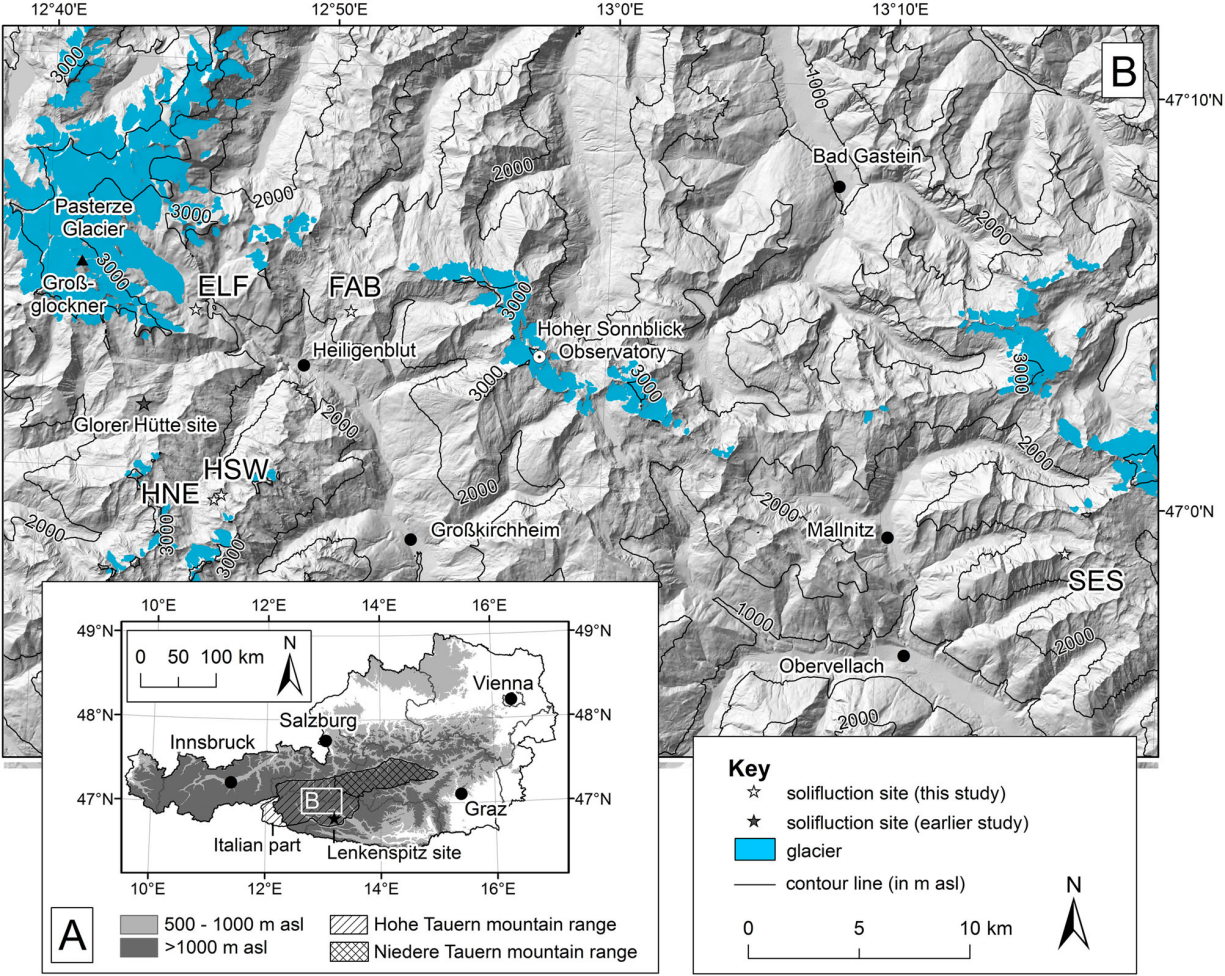
Location of the five solifluction monitoring sites in the Hohe Tauern range, Austria: FAB = Fallbichl, ELF = Elisabethfelsen, SES = Seeschartl, HSW = Hinteres Langtal cirque – south-west facing slope, and HNE = Hinteres Langtal cirque – north-east facing slope; dark grey lines in A indicate the borders of the national provinces of Austria; contour lines in B are 1000 m equidistant

Quantification of solifluction rates has been done in polar and subpolar regions in preference to mid-latitude and tropical regions. These latter regions have received less attention in the past, as revealed by Matusoka (2001) in his global review of solifluction rates, processes and landforms. Some regional exceptions exist, such as the Japanese Alps (e.g. Matsuoka 1998a; 1998b), the Rocky Mountains (e.g. Benedict 1970; Smith 1992) and the Swiss Alps. In the latter region, research on the distribution of solifluction landforms and the quantification of past and present solifluction rates has a long tradition (e.g. Furrer 1954; 1965; Gamper 1983; 1987; Matsuoka et al. 1997; 2011; Krummenacher et al. 1998). In the Austrian Alps farther to the east of the Swiss Alps, solifluction landform detection and process monitoring has been carried out by, for example, Höllermann (1967), Stingl (1969), Stocker (1973; 1984), and Höfner (1993). Detailed solifluction monitoring in Austria focussed particularly on one study area in central Austria, namely the Glorer Hütte site (Fig. 1B), where a monitoring plot for solifluction movements was initiated in 1985 (Veit & Höfner 1993; Veit et al. 1995) and extended in 1994 (Jaesche 1999; Jaesche et al. 2003). Research activities at the site decreased substantially in recent years, due to personnel changes (Stingl et al. 2010) (J.C. Otto, personal communication 2017). Apart from this site, little is known about recent solifluction movement rates in other areas of the Austrian Alps. Furthermore, nothing is known about regional similarities or differences regarding solifluction rates and respective drivers in Austria, because solifluction measurements have not been performed previously at several sites distributed over a larger area at the same time. The study on which this article is based aimed to reduce this gap.

Solifluction rates and related environmental conditions were analysed at five selected solifluction lobes in different alpine settings in central Austria over a period of four years (2006–2010). The research questions addressed in this article are: What are the present solifluction rates in central Austria? What type of solifluction mode – needle-ice creep, frost creep, gelifluction, or plug-like flow – is the dominant solifluction component? Is there a correlation between the movement behaviour in the different study sites? What are the driving factors for solifluction in the region? Therefore, the main aims of the study were: (1) to quantify recent solifluction rates at different periglacial sites in the Austria Alps; (2) to reveal relationships between solifluction and environmental controls at the studied sites, and (3) to contribute to knowledge about the relationship between solifluction rates and environmental conditions at a regional scale in a mid-latitude mountain region.

## The studied landforms

All studied solifluction lobes were located in the central part of the Hohe Tauern mountain range, central Austria, in a 38 km (west–east) by 11 km (north–south) wide region (Fig. 1A). Solifluction monitoring was performed at five sites: Fallbichl (FAB), Elisabethfelsen (ELF), Seeschartl (SES), a south-west facing slope at the Hinteres Langtal cirque (HSW), and a north-east facing slope at the Hinteres Langtal cirque (HNE). The selection of the five solifluction lobes was based on the following criteria:

- a regional distribution of the studied solifluction landforms and hence, to some extent, a region-wide consideration of the central part of the Hohe Tauern mountain range
- the morphological evidence that solifluction acted on these slopes at least sometimes in the past (only solifluction landforms were studied)
- the possibility to assess local topoclimatic effects by studying in one valley two comparable solifluction lobes orientated on opposed valley slopes (HWS and HNE)
- the possibility during fieldwork to use synergies with the permafrost and periglacial monitoring network established at nine sites in the Hohe Tauern and Niedere Tauern mountain ranges (Fig. 1A) by the Univerity of Graz and the Graz University of Technology (Kellerer-Pirklbauer & Kaufmann 2012; Kellerer-Pirklbauer 2016).

The Hohe Tauern mountain range is a 140 km long (east–west) and 70 km wide (north–south) and constitutes the geographical focus of the Eastern Alps, covering a total area of 6070 km^2^ – 5380 km^2^ in Austria and 690 km^2^ in Italy. Most of the highest peaks of Austria are located in this mountain range, including the highest summit of Austria, Großglockner (3798 m a.s.l.) (Fig. 1B). Some 150 km^2^ of the Austrian part of the Hohe Tauern mountain range is glaciated (Fischer et al. 2015) and comprises the largest glacier in Austria, the Pasterze Glacier (17 km^2^) (Fig. 1B) (Kaufmann et al. 2015). According to a regional permafrost model (Boeckli et al. 2012) and as is evident from numerous active rock glaciers (Kellerer-Pirklbauer et al. 2012), permafrost influences 10–15% of the mountain range.

**Fig. 2.**
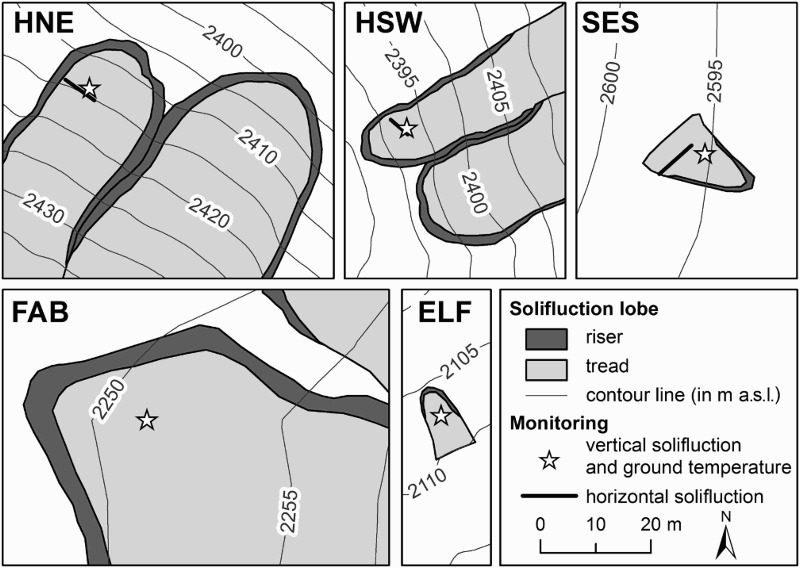
The five solifluction monitoring sites, showing monitoring set-up and their relative positions at the studied solifluction lobes; contour lines (equidistance 5 m) derived from 1 m (HNE, HSW, ELF) and 10 m (FAB, SES) digital elevation models

**Fig. 3.**
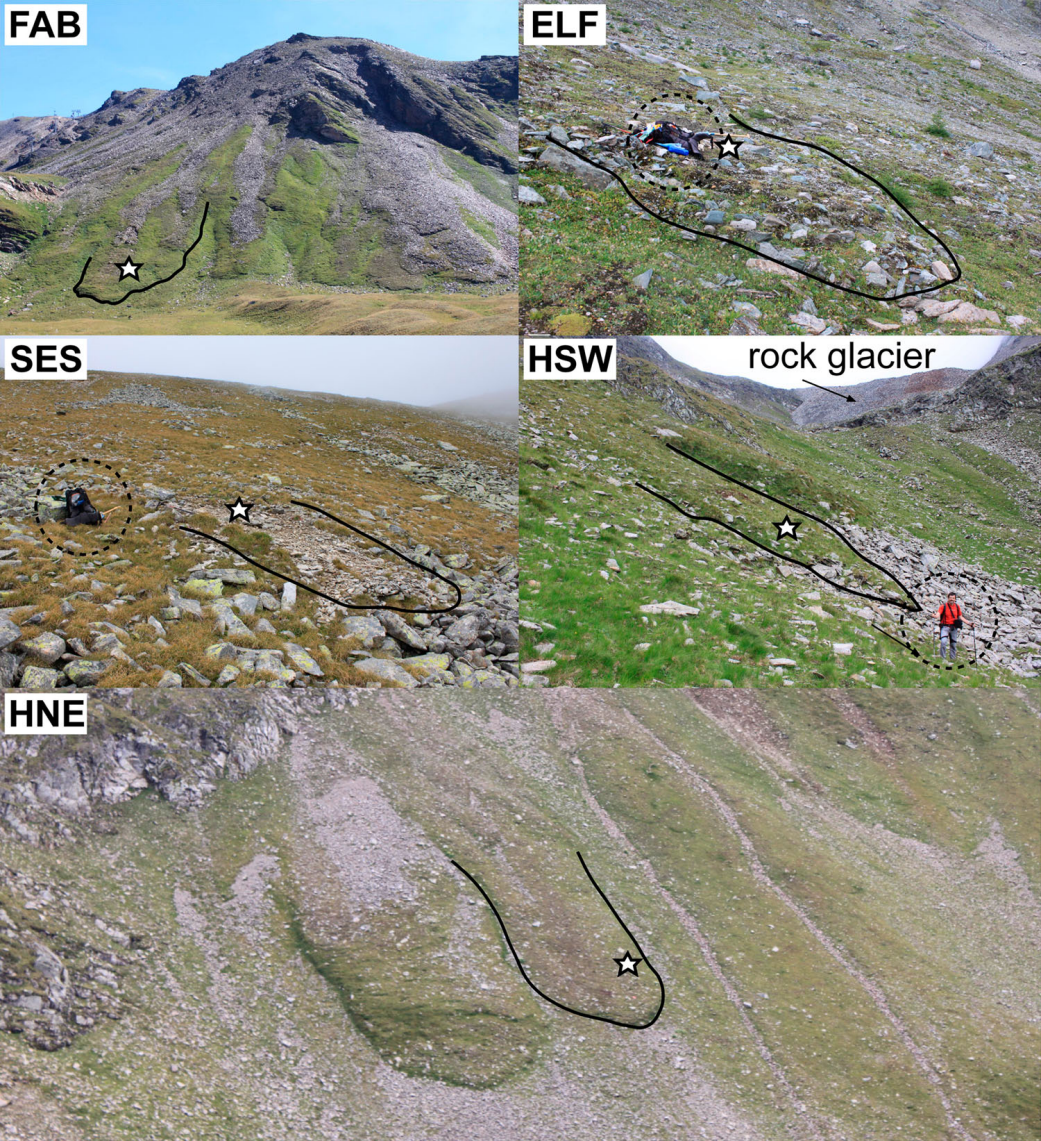
Terrestrial view of the five studied solifluction landforms; star symbols mark the monitoring sites; stippled circles highlight either a backpack or person for scale; (Photos: Kellerer-Pirklbauer, 2006 – ELF, SES, HSW and HNE, and 2007 – FAB)

Solifluction lobes are commonly subdivided in terms of the presence or absence of vegetation at the riser and at the tread (cf. Matsuoka et al. 2005) of the lobe, and this presence or absence is used to differentiate between turf-banked and stone-banked lobes. Turf-banked lobes comprise those with a complete vegetation cover and those that only have vegetation at the riser (Benedict 1970; Matsuoka 2001). Solifluction landforms that are almost identical in size, shape, slope orientation, slope angle, vegetation, and material do not exist in the five study areas. Therefore, the studied lobes vary in their characteristics. The selected solifluction lobes covered areas in the range of 70–6474 m^2^, were 18–251 m long, and terminated at elevations of 2105–2593 m a.s.l. Mean slope angles of the landforms were 18.5–35.5° and the frontal slope (riser) was 45–90 cm high (Table 1). The two solifluction lobes at the monitoring sites SES and ELF were substantially smaller (70–71 m^2^) compared with the other three lobes (616–6474 m^2^). These two smaller lobes were classified as stone-banked lobes, while the large ones were classified as turf-banked lobes. Mica schist is the dominant parent rock material at three of the sites (FAB, HSW, and HNE). Orthogneiss and its weathering products are dominant at SES. Lastly, basal till consisting of different types of metamorphic rocks occurs at FAB. Figure 2 shows details of the studied landforms and their monitoring locations. Figure 3 shows terrestrial views of all five sites.

**Table 1.** Key data of the five solifluction lobes where monitoring was performed

Location of lobe	Area (m^2^)	Length (m)	Max. width (m)	Elevation at front (m a.s.l.)	Mean slope (°)	Aspect (class)	Frontal height of lobe (riser) (cm)	Vegetation cover		Landform*	Geology**
								riser	tread		
Fallbichl	6474	251	67	2247	29.5	NW	90	closed	closed	TBL	CMS
Elisabethfelsen	71	18	9	2105	22.2	NNW	50	sparse	sparse	SBL	MR
Seeschartl	70	18	12	2593	18.5	SE	45	sparse	no	SBL	OG
Hinteres Langtal cirque - south-west facing slope	616	74	22	2391	34.6	SW	60	sparse	sparse	TBL	MS
Hinteres Langtal cirque - north-east facing slope	1661	105	44	2406	35.3	NNE	55	sparse	sparse	TBL	MS

Notes: * TBL = turf-banked lobe; SBL = stone-banked lobe; ** CMS = calcareous mica schist, MR = basal till consisting of different types of metamorphic rocks, OG = orthogneiss, MS = mica schist

## Methods and data analysis

### Solifluction rates: data acquisition

The five monitoring sites were close to the lower end of the respective solifluction lobes, with the exception of the small lobes at ELF and SES, where research was carried out in the central part of the tread (Figs. 2 and 3). Table 2 gives an overview of the monitoring sites with codes, topographic parameters, and monitoring information, including available data series. As shown in Table 2, the five study sites varied in elevation (by 488 m, range 2107–2595 m a.s.l.), in slope (by 20°, range 14–34°), and in aspect (by 294°, range 28–322°). Thus, the five sites covered a wide range of different local topoclimatic conditions.

**Table 2.** Site description, monitoring set-up and data series at the five solifluction monitoring sites

Location of monitoring site	Code	Elevation (m a.s.l.)	Slope (°)	Aspect (°)	Solifluction monitoring*			Ground temperature data series**	
					period (MMYY)	vertical(depth, cm)	horizontal (length, cm)	period (MMYY)	sensor depths (cm)
Fallbichl	FAB	2251	14	285	0907–0810	FLT (50)	nd***	0907–0810	0,10,75
Elisabethfelsen	ELF	2107	21	322	0906–0810	FLT (50)	nd	0906–1107 0909–0810	0,10,55
Seeschartl	SES	2595	18	96	0906–0810	RBC (42)	CSL (550)	0906–0807 0708–0810	0,10,70
Hinteres Langtal cirque – south-west facing slope	HSW	2393	32	253	0906–0810	RBC (60)	CSL (650)	0906–0810	0,10,40
Hinteres Langtal cirque – north-east facing slope	HNE	2415	34	28	0906–0810	RBC (50)	CSL (700)	0906–0810	0,10,40

Notes: * RBC = Rudberg columns, FLT = flexible tubes (profile depth in cm), CSL = coloured sprayed lines (length of line in cm); ‘vertical’ = displacement measured perpendicular to the surface, ‘horizontal’ = surface-parallel displacement measurements; ** Data gaps for ELF and SES; *** nd = no data

I measured solifluction rates at and near the surface in two different ways by applying non-electronic methods, each year between late August and early September in the period 2006–1010. First, solifluction was quantified at all five sites by near-surface markers used as vertical profiles inserted perpendicular to the ground surface with profile depths of 0.4–0.6 m. With this method, information on movement changes with depth was provided. Second, displacement at ground surface was monitored at cross-sections orientated perpendicular to the slope by painted lines (e.g. Price 1973; Matsuoka 2001) at three of the five sites where vegetation was sparse.

**Fig. 4.**
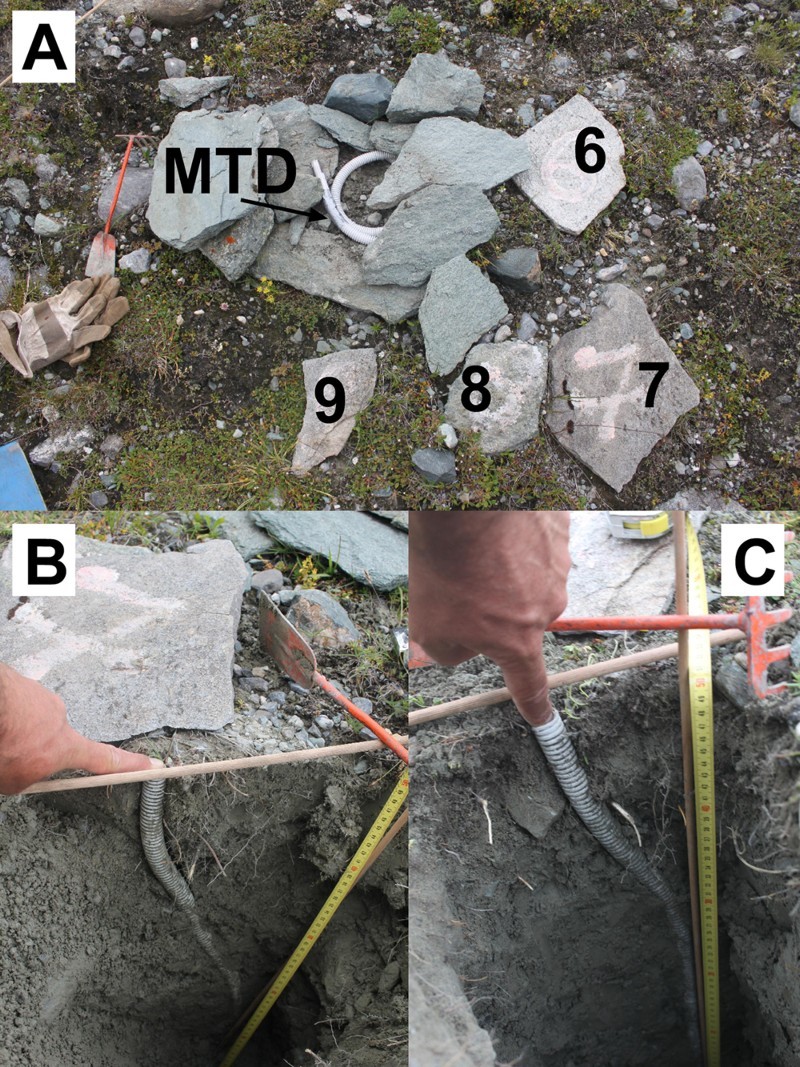
Excavation of flexible tubes at the solifluction site ELF; (A) the location of the four different flexible tubes (labelled 6, 7, 8, and 9) inserted into the ground between 2006 and 2009, and the location of the 3-channel miniature temperature datalogger (MTD); gloves for scale; (B) and (C) the vertical movement profiles for the periods 2007–2010 and 2009–2010, respectively (Photos: Kellerer-Pirklbauer, 2010)

**Fig. 5.**
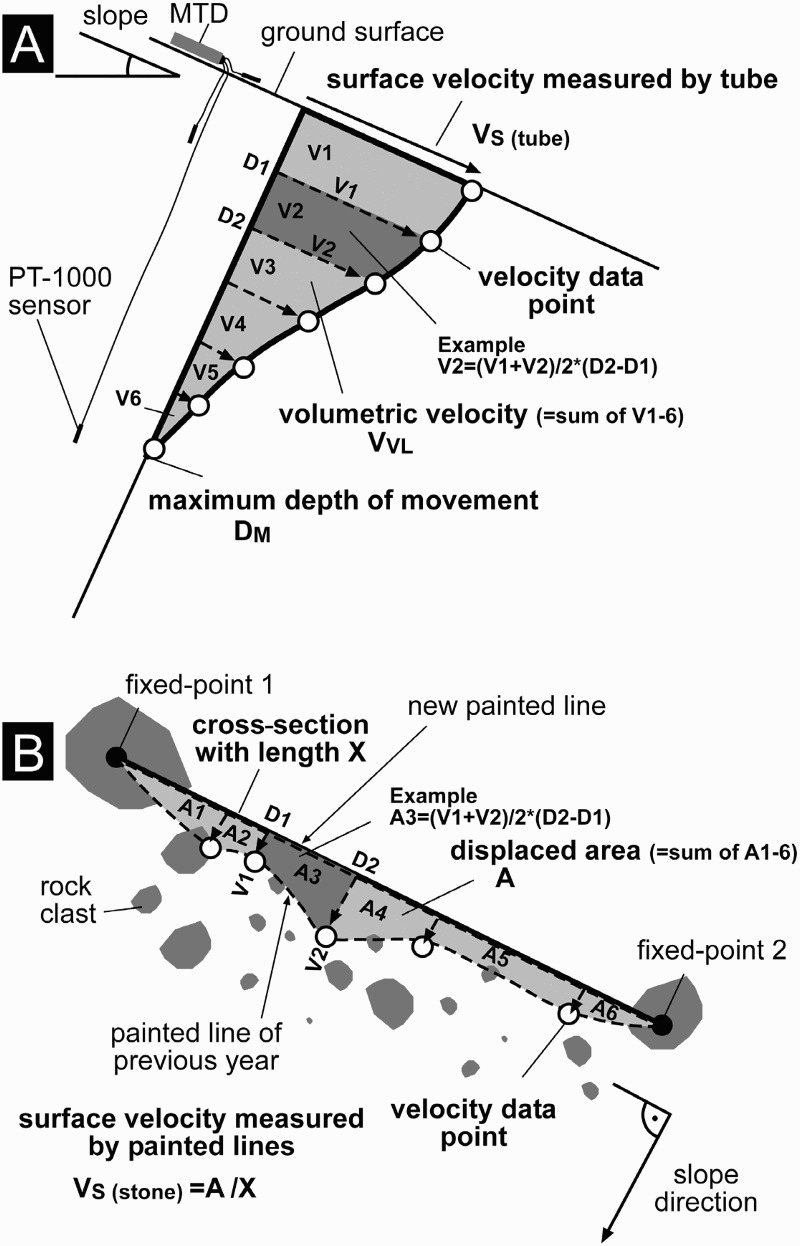
Solifluction movement parameters relevant for (A) near-surface vertical soil profiles (perpendicular to the ground surface) and (B) cross-sections at the surface of solifluction monitoring site, with equations used; (A) modified from Matsuoka (2001, 117), with the position of a miniature temperature datalogger (MTD) with its three temperature sensors shown schematically (for specific sensor depths, see Table 2)

To measure the cumulative deformation of near-surface markers during the four-year monitoring period, either 2 cm long inflexible tube pieces (Rudberg columns (RBCs) (Rudberg 1962)) or up to 50 cm long flexible tubes (FLTs) (Matsuoka 2001) were used (Table 2). According to Matsuoka (2001), the use of RBCs and FLTs yield similar velocities on the same slope and therefore the results can be considered comparable with each other. Each year between late August and early September, fieldwork was carried out at the solifluction moitoring sites. RBCs or FLTs were placed into the ground at the solifluction lobes. Boreholes up to 60 cm deep (Table 2) and 2 cm wide were first manually drilled perpendicular to the surface using a soil auger. Subsequently an RBC or FLT with a diameter of 2 cm was placed carefully into each of the boreholes. The auger had the same diameter as the RBCs and FLTs, and therefore kept soil disturbance to a minimum (cf. Smith 1992). During the placing, a wooden stick that fitted precisely inside the tubes or tube pieces was used to keep the RBC or FLT in a straight line. After installation of the RBC or FLT, the wooden stick was carefully removed. This installation was done annually between 2006 and 2009, except at FAB, where monitoring was initiated in 2007. Continuous monitoring, such as that done by, for example, Berthling et al. (2000), who used differential GPS, and Harris et al. (2008), who used a steel construction, was not feasible in the study reported here. Each year, the new borehole was positioned at a distance of c.10–20 cm from the earlier one (see the example in Fig 4A, with the position sites of the flexible tube in 2006, 2007, 2008, and 2009) to avoid artificial disturbance of the previously installed RBCs or FLTs. Between the 19th and 24th of August 2010 all vertical profiles at the five solifluction sites were excavated. Fig. 4B and 4C show examples of excavation work at ELF. After excavation, the cumulative surface and subsurface movement of 1–4 years was carefully measured at all profiles, as shown in Fig. 4B and 4C and in Fig. 5A.

At three sites, SES, HSW, and HNE, surface displacement at 5.5–7.0 m wide cross-sections at the surface perpendicular to the slope direction was additionally monitored. The cross-sections had two fixed points at the surface of boulders at both ends of a straight line. Given the large sizes of the studied solifluction lobes, it was not feasible to consider their entire width at HNE and HSW. Therefore, it was presumed that the ‘fixed’ points at HNE and HSW similarly moved slightly downward during the observation period. The fixed points at SES were one large block at the right-hand (south-west) edge of the lobe that showed no obvious signs of movement during the observation period and one large block in the central part of the lobe (Fig. 2). Given the large size of the boulders selected as fixed points, it was initially assumed that the boulders acted as local solifluction brakes. The original assumption was later supported by the painted-line data, which showed downslope convexity between the two points at all three sites. The solifluction rates quantified using the painted-line approach must therefore be seen as minimum values and it is conceivablethat the solifluction rates were slightly higher.

Between late August and early September in each of the years 2006–2010 inclusive, a string line was stretched between the two fixed points. The soil and rock material along the painted line was painted a different colour each year to avoid confusion in the following years. As a next step, the shift between the new painted line and rocks that had been painted in the previous year was measured. The shifted area was determined and divided by the profile length to reveal the mean surface velocity measured using the painted lines for the respective cross-sections (see Fig. 5B for graphical explanation).

### Solifluction rates: data analysis

Solifluction movement is defined by the three parameters surface velocity (V_S_), volumetric velocity (V_VL_), and maximum depth of movement (D_M_) (Matsuoka 2001) (Fig. 5). V_S_ represents the mean annual downslope movement at the surface. V_VL_ is defined as the annual soil volume passing through a unit width (1 cm) in the dimension of cm^3^ cm^−1^ yr^−1^. D_M_ represents the base of the detectable movement. In the study reported here, V_S_ was calculated in two different ways: first, by using the displacement data derived from the tops of the RBC or FLT (V_S (tube)_), and second, by calculating the mean value of the surface velocity values measured at the 5.5–7.0 m wide cross-sections with the colour painted lines (V_S (stone)_). Therefore, both of the V_S_ approaches described by Matsuoka (1998a; 2001) were applied. Matsuoka (1998a; 2001) has pointed out that alpine slopes in mid-latitude mountain areas show substantially higher V_S (stone)_ rates compared with V_S (tube)_ rates because only the former responds to needle-ice creep.

For all four parameters (V_S (tube)_, V_S (stone)_, V_VL_, and D_M_) annual values were computed. The depth of movement (D_M_) was measured in 5 cm increments. A higher resolution in the quantification of the D_M_ did not seem reasonable because of the gentle break of slope in the vertical RBC and FLT profiles (cf. Fig. 4C). Surface velocity rates based on tubes (V_S (tube)_) was measured using 0.5 cm increments. Figure 5 shows how the four solifluction movement parameters were derived.

### Environmental conditions: air temperature

The mean annual air temperature (MAAT) for the period 1 September 2006 to 31 August 2010 was calculated for each solifluction monitoring site by using data from nearby automatic weather stations and a lapse rate of 6 °C km^−1^ (Matsuoka 2001). For FAB and ELF, data from the automatic weather station Margaritze (run by VERBUND Hydro Power GmbH) at 2070 m a.s.l. were used. The station was located 1 km north-east of ELF and 6 km west of FAB. For SES, HSW, and HNE, data from two automatic weather stations run by the Department of Geography and Regional Science, University of Graz, were available. For SES, the relevant station was 1 km north-east of the solifluction site and at 2603 m a.s.l. For the two neighbouring sites HSW and HNE, the relevant weather station was respectively located 0.6 km and 0.8 km north-east and at 2655 m a.s.l. Direct snow data from the studied landforms were missing. In order to describe the general snow conditions between 2006 and 2010, daily snow-depth data were used from a meteorological observatory located in the central part of the study region at Hoher Sonnblick (Fig. 1B).

### Environmental conditions: soil texture

At each of the five studied solifluction lobes, a soil sample was collected for grain-size distribution analysis. Debris samples for sieving (0.5–0.8 kg) were taken from the near-surface layer (c.5 cm depth) at each site. The determination of the grain size distribution followed the requirements of the Austrian normative regulation (ÖNORM) 4412, using conventional wet sieving analysis for grain sizes > 0.063 mm.

### Environmental conditions: ground temperature

Ground temperature was measured during the monitoring period at each solifluction site. Temperature sensors were installed at three different depths, forming vertical profiles (maximum 75 cm) (Table 2). At all sites 3-channel miniature temperature dataloggers (MTD) with three individual PT1000 temperature sensors (GeoPrecision, Model M-Log6) were used (Figs. 4 and 5). The PT1000 sensor nearest to the surface was sheltered from direct solar radiation by thin platy rocks that still allowed rather unhampered air circulation within the voids. According to GeoPrecision GmbH, the used PT1000 temperature sensors in the MTDs had an accuracy of ±0.05 °C and a range of −40 °C to +100 °C. Temperature was logged every 60 minutes. Data gaps existed (Table 2), due to cables damage caused by rodents.

Ground temperature data were analysed regarding ground thermal conditions and for estimating the role of the different solifluction components needle-ice creep, diurnal frost creep, annual frost creep, and gelifluction, since direct data for soil heave or soil moisture were missing. These parameters are summarized in Table 3, which includes their respective abbreviations. The components needle-ice creep and diurnal frost creep were estimated by calculating the number of diurnal freeze-thaw cycles (FTCs) and the number of effective diurnal freeze-thaw cycles (eFTCs) for freezing (Matsuoka 1990). The relevance of the component annual frost creep was assessed by the following proxies: the mean annual ground temperature at the surface (MAGST) and at different depths (MAGT), the annual sum of positive (thawing) degree days per year (TDD), the annual sum of negative (freezing) degree days per year (FDD), and by the depth of frost/thaw penetration (DF/DT). The MAGST was used as an indication of whether a solifluction site was influenced by permafrost or seasonal frost.

**Table 3.** Parameters derived from continuous ground temperature data relevant for different solifluction components

Parameter	Description	Main movement component*	Relevant period for calculation	Considered sensor	Unit
FTC	freeze-thaw cycle (diurnal)	NIC/DFC	1 Sept.-31 Aug.	uppermost	number
eFTC	effective freeze-thaw cycle (diurnal)	NIC/DFC	1 Sept.-31 Aug.	uppermost	number
MAGST/ MAGT	mean annual temperature: surface and at different depths	AFC	1 Sept.-31 Aug.	all three sensors	°C
TDD	sum of positive (thawing) degree days per year	AFC/GF	1 Sept.-31 Aug.	all three sensors	degree days
FDD	sum of negative (freezing) degree days per year	AFC	1 Sept.-31 Aug.	all three sensors	degree days
DF/DT	depth of frost/thaw penetration	AFC	1 Sept.-31 Aug.	all three sensors	cm
ZCP	zero curtain period	GF	spring-summer	uppermost	days
STP	seasonal thawing period	GF	spring-summer	lower two sensors	days
SOD	snow onset date	AFC/DFC	Autumn	uppermost	day of year
MGST-O	mean ground surface temperatures in October	AFC/DFC	October	uppermost	°C
MGST-CM	mean ground surface temperatures of coldest month	AFC/DFC	1 Sept.-31 Aug.	uppermost	°C
MGST-JJ	mean ground surface temperatures in June/July	GF	June/July	uppermost	°C
MGST-WM	mean ground surface temperatures of warmest month	GF	1 Sept.-31 Aug.	uppermost	°C

Note: *NIC = needle-ice creep, DFC = diurnal frost creep (shallow thermal influence), AFC = annual frost creep (deep thermal influence), GF = gelifluction (as defined by Matsuoka 2001)

Matsuoka (2001) has noted that the freeze-thaw penetration limits the extent to which solifluction can occur. The depth of frost (DF) or thaw (DT) penetration was therefore estimated as follows. Mean ground temperature profiles (using the data from the three different sensors) (Table 2) were calculated based on monthly values, where the mean annual temperature, the mean of the coldest month, and the mean of the hottest month were plotted against depth. According to van Everdingen (1985), the linear trends of the temperatures of the hottest and warmest months will tend to meet at a point where the annual amplitude is 0 °C. Therefore, by applying this linear trend analysis approach, it was possible to estimate the depth of frost or thaw penetration for each year and consequently also the existence of permafrost.

The relevance of the solifluction component gelifluction was assessed by calculating the duration of the zero curtain period (ZCP) at the surface and the duration of the seasonal thawing period (STP) at the two sensors below the surface. The ZCP value was used as an indicator of water availability during the spring melting season and hence as a proxy for water saturation of the soil. The ZCP was defined as the number of days during spring when the mean daily temperature was 0 °C at the ground surface. The onset of the ZCP was when the frozen ground surface warmed to 0 °C by meltwater percolation or by strong rain-on-snow events. The onset of the ZCP indicated the possible onset of ground thawing from above. The end of the ZCP was defined by the melt-out date (i.e. the time when the snow cover had completely melted and no further meltwater was transferred to the ground, thus allowing the ground surface to warm above 0 °C). The STP was defined as the number of days in spring and early summer when the maximum daily temperature was between 0 °C and +2 °C. The upper threshold of +2 °C was based on Matsuoka (1990), who defined an effective freeze-thaw cycle with −2 °C for freezing and +2 °C for thawing. A short STP indicated rapid heating and hence suggested a lack of soil moisture, while a long STP indicated the slow release of latent heat by thawing of ground ice and hence the availability of soil moisture for gelifluction.

Additionally, the snow onset date in autumn (SOD), the mean ground surface temperature in October (MGST-O), the mean ground surface temperatures of coldest month (MGST-CM), the mean ground surface temperatures of June/July (MGST-JJ), and the mean ground surface temperatures of warmest month (MGST-WM) were calculated using the ground temperature data (Table 3). Whereas the first three parameters SOD, MGST-O, and MGST-CM were relevant for autumn and winter cooling, the latter two parameters, MGST-JJ and MGST-WM, are considered important for the thawing rate of the ground and the active layer thickness (Veit et al. 1995; Matsuoka 2011). Fieldwork at the solifluction sites was done each year between late August to early September. Therefore, the annual period relevant for the calculation of the annual parameters listed in Table 3 was 1 September to 31 August of the following year.

## Results

### Solifluction movement

**Fig. 6.**
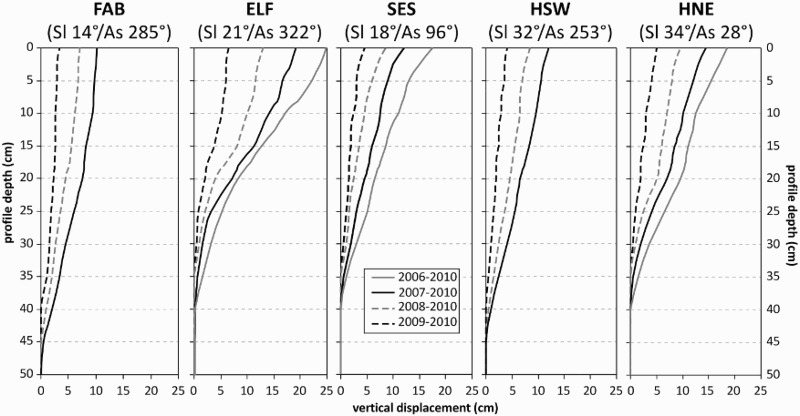
Annual velocity profiles based on the cumulative subsurface movement measured with flexible tubes (FAB, ELF) or Rudberg columns (SES, HSW, and HNE); no data for the period 2006–2010 at the two sites FAB and HSW; slope gradient (Sl) and aspect (As) are indicated for each site

Figure 6 shows the annual velocity profiles based on the cumulative subsurface movement. The annual and mean annual values of V_S (tube)_, V_S (stone)_, V_VL_, and D_M_ are summarized in Table 4. The monitoring results indicate downslope concavity of the velocity profiles, particularly at SES (the highest above sea level). The downslope concavity was less pronounced for ELF (stone-banked lobe), HSW, and HNE (both turf-banked lobes). At FAB, which was completely covered in vegetation in the form of an alpine meadow (Fig. 3), no downslope concavity was observed in the movement profiles. Instead, a slight tendency towards downslope convexity was seen.

**Table 4.** Summarized results for surface velocity measured near the surface using Rudberg columns (RBCs) or flexible tubes (FLTs) (V_S (tube)_), surface velocity measured directly at the surface using painted lines (V_S (stone)_), volumetric velocity (V_VL_), and maximum depth of movement (D_M_) (nd = no data)

Site*	Year	V_S (tube)_	V_S (stone)_	V_VL_	D_M_
		(cm yr^−1^)	(cm yr^−1^)	(cm³ cm^−1^ yr^−1^)	(cm)
FAB	07-08	3.0	nd	103	45
	08-09	4.0	nd	96	40
	09-10	3.5	nd	83	35
	*mean*	*3.5*	* *	*94.0*	*40.0*
ELF	06-07	5.5	nd	94	35
	07-08	6.0	nd	101	35
	08-09	6.5	nd	109	30
	09-10	6.5	nd	105	30
	*mean*	*6.1*	* *	*102.3*	*32.5*
SES	06-07	5.5	10.1	91	45
	07-08	3.5	8.4	77	45
	08-09	4.0	7.7	53	35
	09-10	4.5	9.6	63	30
	*mean*	*4.4*	*8.9*	*71.0*	*38.8*
HSW	06-07	nd	5.4	nd	nd
	07-08	3.5	5.2	91	40
	08-09	4.5	6.5	100	40
	09-10	4.0	7.6	77	35
	*mean*	*4.0*	*6.2*	*89.3*	*38.3*
HNE	06-07	4.0	5.8	91	35
	07-08	5.0	8.1	86	35
	08-09	4.5	6.6	93	30
	09-10	5.0	6.8	77	30
	*mean*	*4.6*	*6.8*	*86.8*	*32.5*

Notes: *FAB = Fallbichl; ELF = Elisabethfelsen; SES = Seeschartl; HSW = Hinteres Langtal cirque – south-west facing slope; HNE = Hinteres Langtal cirque – north-east facing slope

**Fig. 7.**
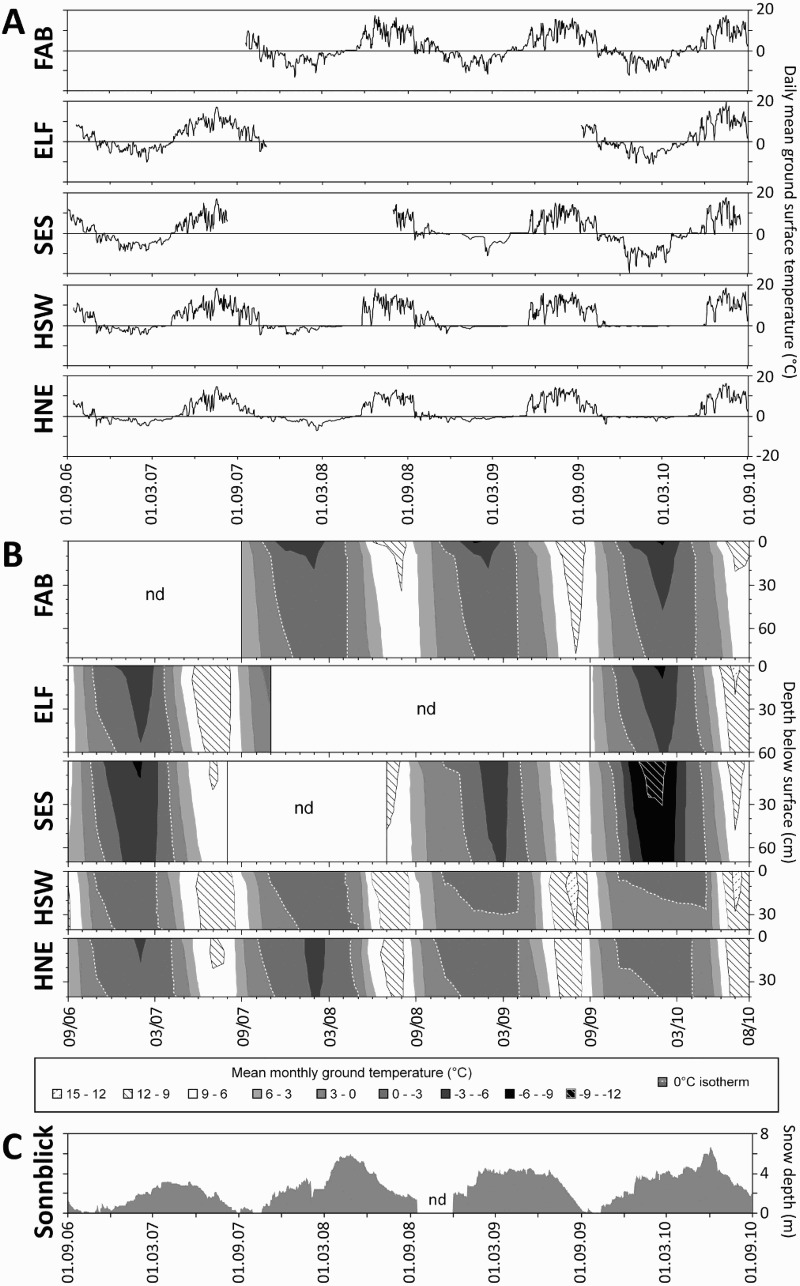
Mean daily ground surface temperature (A) and subsurface isotherms (3 °C intervals) based on mean monthly values (B) at the five solifluction sites between September 2006 and August 2010; dashed line marks the 0 °C isotherm; nd = no data; daily depth of snow at Hoher Sonnblick Observatory shown in C, indicating general snow conditions

The surface velocities derived from the tops of the Rudberg columns (RBCs) or flexible tubes (FLTs) (V_S (tube)_) suggest relatively uniform annual movement rates at each site during the monitoring period. The mean V_S (tube)_ varied between 3.5 cm yr^−1^ at FAB and 6.1 cm yr^−1^ at ELF. At the remaining three sites, the mean annual value was in the range of 4.0–4.6 cm yr^−1^. The difference between the highest and lowest measured annual V_S (tube)_ value varied between 2.0 (at SES) and 1.0 cm yr^−1^ (at the remaining four sites). The highest V_S (tube)_ value was measured at the different sites during different years. By contrast, the lowest V_S (tube)_ value was measured at all five sites, either for the measurement year 2006–2007 (which had snow-poor winter as judged from the general snow conditions during that year in the region) (Fig. 7C) or for 2007–2008 (which had a ‘normal snow’ winter).

The surface velocity derived from painted lines (V_S (stone)_) was calculated at SES, HSW, and HNE. The mean V_S (stone)_ was 6.2 cm yr^−1^ at HSW, 6.8 cm yr^−1^ at HNE, and 8.9 cm yr^−1^ at SES. Similar to the V_S (tube)_ values, the pattern for the highest and lowest values was not synchronous at the five sites. The highest displacement rates were measured at SES during the first monitoring year, at HNE during the second monitoring year, and at HSW during the fourth monitoring year. The difference between the highest and lowest measured annual V_S (stone)_ value at all three sites was in the order of 2.3–2.4 cm yr^−1^.

The mean values of the volumetric velocity (V_VL_) at the five sites ranged from 71 cm^3^ cm^−1^ yr^−1^ at SES to 102 cm^3^ cm^−1^ yr^−1^ at ELF. The mean values of V_VL_ at the remaining three sites, FAB, HSW, and HNE, were comparable, with 87–94 cm^3^ cm^−1^ yr^−1^. During three of the four monitoring years, the highest V_VL_ value was measured at ELF, with the exception of 2007–2008. The range between the highest and lowest V_VL_ value during the observation period was between 15 cm^3^ cm^−1^ yr^−1^ at ELF and 38 cm^3^ cm^−1^ yr^−1^ at SES.

The mean D_M_ ranged from 32.5 cm at HNE and ELF to 40 cm at FAB (Table 4). The interannual variability of D_M_ was between 5 cm at ELF, HSW, and HNE, 10 cm at FAB, and 15 cm at SES. The highest recorded value for D_M_ was 45 cm, which was measured once at FAB (2007–2008) and twice at SES (2006–2007, 2007–2008). By contrast, the lowest D_M_ was 30 cm, measured twice at ELF (2008–2009, 2009–2010), once at SES (2009–2010), and twice at HNE (2008–2009, 2009–2010).

### Environmental conditions

The MAAT during the observation period at the five monitoring sites was positive (range 0.2–2.3 °C) in four cases and only negative (−1.5 °C) at SES. By far the highest MAAT was recorded at all five sites in the first monitoring year 2006–2007 (c.1.5 °C warmer compared with the four-year mean value). By contrast, the coldest monitoring year at all sites was 2009–2010 (Table 5). Based on the MAAT, it can be confirmed that seasonal frost existed at two sites, FAB and ELF, and that permafrost might have occurred to a minor extent at HSW and HNE (Table 6). However, permafrost conditions only dominated at the highest monitoring site, SES (warm), during the observation period.

**Table 5.** Mean annual air temperature (MAAT) at the five solifluction monitoring sites during the four-year period 1 September 2006 to 31 August 2010, as estimated from nearby automatic weather stations

Site*	MAAT (°C)				
	2006–2007	2007–2008	2008–2009	2009–2010	Mean
FAB	2.9	1.1	1.0	0.6	1.4
ELF	3.7	1.9	1.9	1.5	2.3
SES	0.0	-1.9	-1.9	-2.3	-1.5
HSW	1.9	-0.1	-0.2	-0.3	0.3
HNE	1.8	-0.2	-0.3	-0.4	0.2

Notes: *FAB = Fallbichl; ELF = Elisabethfelsen; SES = Seeschartl; HSW = Hinteres Langtal cirque – south-west facing slope; HNE = Hinteres Langtal cirque – north-east facing slope

**Table 6.** Grain size distribution, soil texture, frost susceptibility, and frost type at the five solifluction monitoring sites

Site*	Grain size (%)		Soil texture	Frost	Frost type***
	Sand	silt-clay		susceptibility**	
FAB	90.6	9.4	sand	intermediate	SF
ELF	79.3	20.7	loamy sand	susceptible	SF
SES	84.3	15.7	loamy sand	susceptible	SF/WPF****
HSW	95.1	4.9	sand	little-susceptible	SF
HNE	95.2	4.8	sand	little-susceptible	SF

Notes: *FAB = Fallbichl; ELF = Elisabethfelsen; SES = Seeschartl; HSW = Hinteres Langtal cirque – south-west facing slope; HNE = Hinteres Langtal cirque – north-east facing slope; ** frost susceptibility as judged from data by Kapler (1974); *** frost type (based on MAAT values) – WPF = warm permafrost, SF = seasonal frost; **** in cold winters WPF conditions, otherwise SF

Soil sieving results indicated a dominance of sand (79.3–95.1%) for all five soil samples, with minor contents of clay and silt (Table 6). The highest sand content was quantified for the two samples at HSW and HNE (c.95%). By contrast, the highest contents of clay and silt were revealed for the samples at ELF (20.7%) and SES (15.7). The soil texture of the analysed soils was therefore either loamy sand (ELF and SES) or sand (FAB, HSW, and HNE). According to Kapler (1974), a silt-clay content of c.5% corresponds to the boundary between frost-susceptible soils and soils not susceptible to frost. Therefore, the soils at HSW and HNE were little susceptible to frost heave. By contrast, the soils at ELF and SES were frost-susceptible. The soil at FAB may be regarded as having been intermediate frost-susceptible.

A summary of the calculated ground temperature derived environmental parameters for each site is given in Table 7. Figure 7A and 7B show the mean daily ground surface temperatures and the subsurface isotherms based on mean monthly data for all five sites. The results indicate high intersite and interannual variabilities. In general, autumn cooling in the subsurface was slower than spring warming, as shown in Figure 7B. The average MAGST was positive at all five sites, ranging from 0.7 °C (SES) to 3.3 °C (HSW). Only once was a negative MAGST value determined at SES (in 2009–2010), due to very cool winter temperatures (Fig. 7A). The average MAGT at depths of 10 cm and 40–75 cm were in the range of 0.9–3.7 °C and 1.0–3.8 °C respectively. The highest average numbers of diurnal FTCs (70.3) and eFTCs (14.7) were calculated for FAB. The second highest FTC and eFTC values were calculated for SES. By contrast, the lowest mean FTC value (33.0) and eFTC value (2.0) were recorded for HNE.

**Table 7.** Summary of environmental parameters derived from continuous ground temperature data (for parameter abbreviations see Table 3)

Site*	Year	FTC	eFTC	MAGST	TDD-0	FDD-0	DF (cm)	ZCP	STP	STP	SOD	MGST	MGST	MGST	MGST
		(n)	(n)	(°C)	(°days)	(°days)		(days)	(days)	(days)	(day of	O	CM	JJ	WM
		0 cm	0 cm	0 cm	0 cm	0 cm		0 cm	10 cm	40-75 cm	year)	(°C)	(°C)	(°C)	(°C)
FAB	07-08	54	12	1.3	1097	-648	115	23	27	24	na***	1.5	-5.2	9.2	10.3
	08-09	84	18	1.8	1274	-606	102	4	42	49	na	3.0	-6.4	8.5	11.7
	09-10	73	14	1.6	1266	-679	115	1	28	36	na	1.9	-6.5	9.7	11.7
	*mean*	*70.3*	*14.7*	*1.6*	*1212*	*-644*	*111*	*9*	*32*	*36*	na	2.1	-6.0	9.1	11.2
ELF	06-07	37	1	2.6	1389	-497	164	0	2	14	na	3.2	-5.4	10.5	11.0
	07-08	nd**	nd	nd	nd	nd	nd	nd	nd	nd	na	0.7	nd	nd	nd
	09-10	38	7	2.0	1373	-666	178	2	5	6	na	1.6	-6.7	11.3	13.3
	*mean*	*37.5*	*4.0*	*2.3*	*1381*	*-581*	*171*	*1*	*4*	*10*	na	*1.9*	*-6.0*	*10.9*	*12.1*
SES	06-07	30	0	1.1	1090	-701	209	7	36	48	na	2.4	-6.3	8.6	9.3
	07-08	nd	nd	nd	nd	nd	nd	nd	nd	nd	na	nd	nd	9.3	11.7
	08-09	72	20	1.6	992	-396	298	38	46	46	na	1.2	-5.1	7.3	10.5
	09-10	70	9	-0.8	937	-1200	443	11	48	40	na	0.9	-10.5	9.1	11.1
	*mean*	*57.3*	*9.7*	*0.7*	*1006*	*-766*	*316*	*18.7*	*43.3*	*44.7*	na	*1.5*	*-7.3*	*8.6*	*10.6*
HSW	06-07	39	2	3.6	1539	-262	58	30	1	15	303	4.6	-2.9	10.2	11.4
	07-08	35	16	2.8	1285	-257	47	28	0	9	na	2.1	-2.5	10.2	11.3
	08-09	53	16	3.3	1328	-119	34	38	1	51	303	3.2	-1.2	9.7	12.2
	09-10	21	1	3.5	1322	-62	26	59	0	64	308	3.0	-0.4	10.7	13.0
	*mean*	*37.0*	*8.8*	*3.3*	*1369*	*-175*	*41*	*38.8*	*0.5*	*34.8*	*287*	*3.3*	*-1.7*	*10.2*	*12.0*
HNE	06-07	39	0	1.9	1039	-356	116	6	21	27	297	1.5	-3.8	8.4	9.2
	07-08	17	0	1.5	961	-404	136	14	15	10	294	0.1	-4.7	8.5	9.7
	08-09	45	8	2.6	1142	-186	60	27	21	19	297	0.2	-1.7	8.9	11.3
	09-10	31	0	2.8	1136	-106	48	23	29	38	309	1.0	-1.0	9.7	12.0
	*mean*	*33.0*	*2.0*	*2.2*	*1070*	*-263*	*90*	*17.5*	*21.5*	*23.5*	*267*	*0.7*	*-2.8*	*8.9*	*10.5*

Notes: *FAB = Fallbichl; ELF = Elisabethfelsen; SES = Seeschartl; HSW = Hinteres Langtal cirque – south-west facing slope; HNE = Hinteres Langtal cirque – north-east facing slope; **nd = no data: **na = not applicable (due to thin snow cover)

At all five sites, the mean value of TDD exceeded the mean value of FDD. The average values for TDD ranged between 1006 (SES) and 1381 (ELF), while the average FDD values ranged from −175 (HSW) to −766 (SES). The mean value for the DF ranged from only 41 cm at HSW to more than 3 m at SES. At SES, a DT value of 328 cm was calculated once for the cold year 2009–2010, which had favourable conditions for permafrost. The duration of the ZCP was longest for the south-west facing site HSW, with 38.8 days yr^−1^on average. SES and HNE revealed similar results, with 17.5–18.7 days yr^−1^. At the two remaining sites, FAB and ELF, the mean ZCP values were below 10 and related to a general thin snow cover at these sites, as revealed by the temperature fluctuations, even in mid-winter (Fig. 7A). Relatively high values of the STP at 10 cm below the surface were quantified for FAB and SES, with mean annual values exceeding 30 days yr^−1^. High values of the STP at 40–75 cm depth were revealed for FAB, SES, and HSW. The shortest STP was calculated for ELF, at 10 cm and at 40–75 cm below the surface (Table 7).

Quantification of the snow onset date (SOD) was not straightforward and only feasible for the two comparatively snow-rich sites, HSW and HNE. High temperature variability and the presence or absence of a distinct winter equilibrium temperature is shown in Fig. 7A. The highest mean temperatures for October (MGST-O) values were commonly measured at HSW (mean 3.3 °C), while the lowest MGST-O values were measured at the site on the opposite side of the valley, HNE (mean 0.7 °C). The coldest MGST-CM was revealed for the highest elevated site, SES, while the warmest MGST-CM value was measured at HSW. The warmest temperatures during summer (i.e. MGST-JJ and MGST-WM) were revealed in both cases for ELF, while the coolest ones were either at SES (MGST-JJ) or at HNE (MGST-NE).

### Relationship between solifluction movement and environmental conditions

Relating the solifluction parameters V_S (tube)_, V_S (stone)_, V_VL_, and D_M_ to the different temperature-derived parameters was performed more qualitatively, due to the short time series that prevented statistical significant correlations. However, the coefficient of determination was high in some cases, but not statistically significant. Therefore, the results presented here regarding correlation between solifluction and ground temperature derived parameters only indicate at most a tendency for correlation and certainly not in a statistically satisfactory manner.

**Fig. 8.**
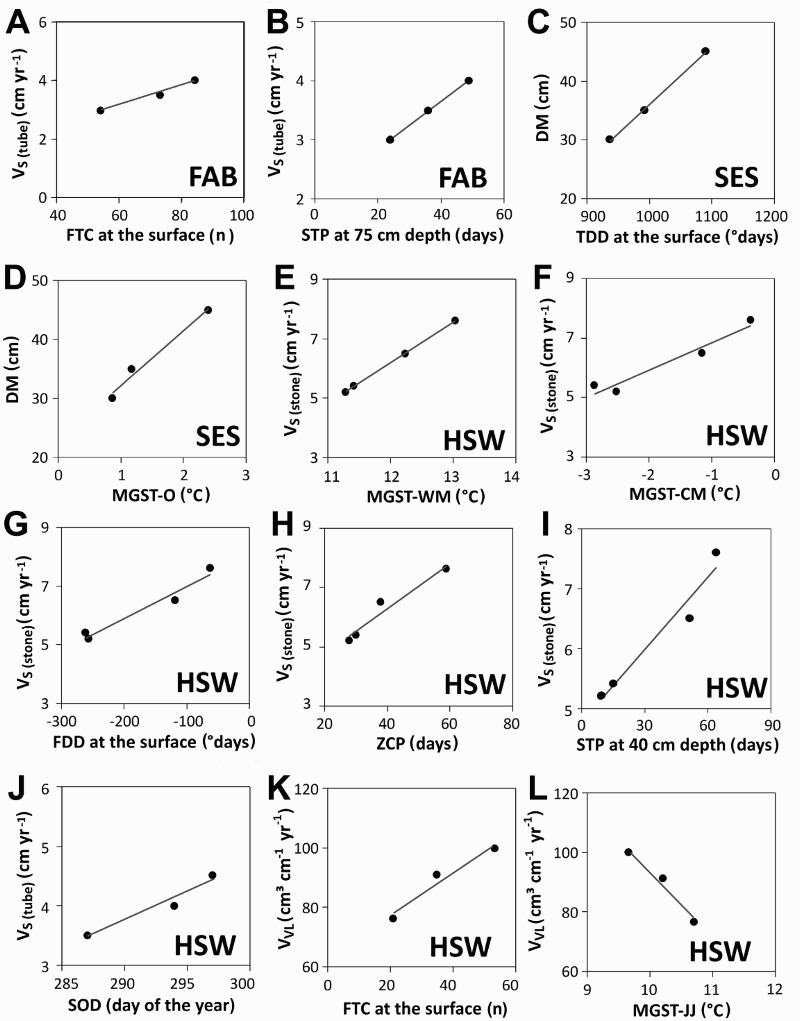
Relationships between movement and different environmental parameters at the different monitoring sites (A) and (B) for site FAB, (C) and (D) for site SES, and (E) to (L) for site HSW, related to the short times series and data gaps; V_S (tube)_ = surface velocity based on Rudberg columns or flexible tubes; V_S (stone)_ = surface velocity based on painted lines; V_VL_ = volumetric velocity; D_M_ = maximum depth of movement; see Table 3 for abbreviations of the different temperature-derived parameters

At FAB, only two meaningful relationships were determined. First, the more FTCs that occurred at the surface and the longer the duration of the STP at 75 cm below the surface, the higher was the surface velocity (Fig. 8A and B). Additionally, two meaningful relationships were revealed for SES (Fig. 8C and 8D). At that site, a positive correlation existed between the TDD at the ground surface and the depth of solifluction movement (D_M_). The higher the TDD value was, the deeper the D_M_ was. Furthermore, the higher the MGST in October (MGST-O) was, the deeper the D_M_ was.

The most informative relationships were found for HSW, with eight interesting relationships (Fig. 8E–L). The milder a winter was (in terms of low FDD values at the surface and in terms of the mean monthly temperature of the coldest month in the year), the higher the surface velocity measured by painted lines (V_S (stone)_) was. Furthermore, the warmer the warmest month in summer (MGST-WM), the higher the V_S (stone)_ was. Finally, the longer the ZCP at the surface and the longer the STP at 40 cm depth below the surface was, the higher the V_S (stone)_ value was. The latter two relationships suggest that gelifluction was relevant at HSW.

Furthermore, the analysis showed that for HSW, the later the snow cover started to form in autumn (SOD), the higher the surface velocity (V_S (tube)_) was. Two interesting relationships were revealed regarding the volumetric velocity: the more FTC occurred at the surface (Fig. 8K) and the cooler the mean ground surface temperature was in June and July (Fig. 8L), the higher the rate of transported mass through solifluction was. These relationships suggest that a late onset of the winter snow cover, frequent freeze-thaw cycles, and gentle warming in early summer are favourable for solifluction at HSW. No satisfactory relationships were detected for ELF and HNE.

## Discussion

### Intersite and interannual variability of solifluction rates

**Fig. 9.**
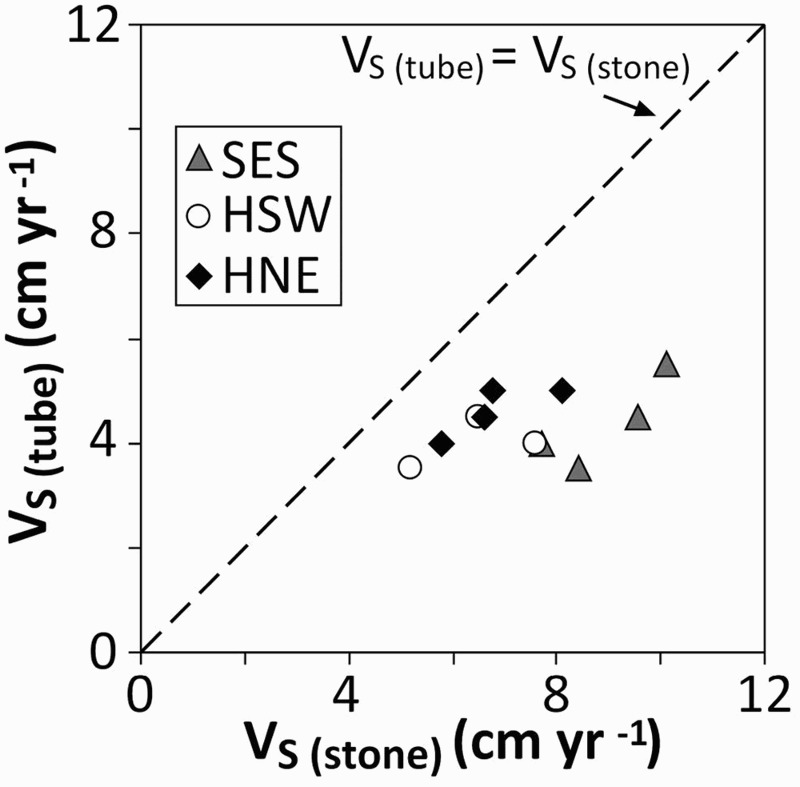
Relationship between annual surface velocities measured by the top of flexible tubes or Rudberg columns (V_S (tube)_) and painted stones (V_S (stone)_)

The measured surface velocities at all three sites with both V_S (stone)_ and V_S (tube)_ data indicate that the painted stones moved substantially faster downslope than did the tops of flexible tubes (Fig. 9). At SES, the V_S (stone)_ was on average 2.0 times faster (range 1.8–2.4) compared with V_S (stone)_. This factor was lower (1.5) at HSW and HNE. This observation confirms the finding of Matsuoka (2001, 122) that surface velocity measured on sparsely vegetated alpine slopes by painted rocks is substantially higher than the surface velocity measured at the same slope at the tops of flexible tubes. Furthermore, bearing in mind that the ‘fixed points’ at both ends of the straight measurement profiles in the study reported here were partly placed at the solifluction lobe (as described in the ‘Methods’ section) and hence possibly moved also slightly downwards, even higher V_S (stone)_ values might be assumed.

Interannual variability in solifluction rates in terms of V_S (tube)_, V_S (stone)_, V_VL_, and D_M_ was not synchronous at the five study sites. This suggests that local conditions (e.g. snow, moisture, and frost) in a given year are more important than general climatic conditions in the region, as revealed by a number of earlier studies from different regions (e.g. Benedict 1970; Gamper 1983; Berthling et al. 2002; Jaesche et al. 2003; Harris et al. 2008; Ridefelt et al. 2009). Interestingly, the site with the lowest V_VL_ values (SES) had the highest interannual variability. This observation highlights the fact that the mass transport rates at SES, a site marginally influenced by permafrost, not only had the highest interannual variability but also the lowest rates of all five sites. By contrast, the mass transport rates by solifluction at ELF were not only the highest ones at all five studied sites but also the most stable ones in terms of interannual variability.

A clear relationship between slope angle and solifluction rates, such as reported in earlier studies (e.g. Price 1973; French 2007), was only partly observed in the study reported here. Matsuoka (1998b) has discussed why the steeper a solifluction monitoring site is, the higher the surface velocity is in general. Matsuoka's observation is also true for the three sites FAB, ELF, SES, for which there was an almost linear relationship between slope angle and V_S (tube)_. However, HNE and HSW showed substantially lower velocities, despite their steep slope gradients. Lower velocities despite steeper slopes were also observed by Matsuoka (1998b), who explained this as due to the influence of other factors, most importantly by the characteristics of the transported material. His study, as well as earlier studies (e.g. Pérez 1988) revealed that small grains tend to move more rapidly. Furthermore, pore size in soils affects the suction force. The rate of frost heave depends linearly on the percentage of silt and clay in the soil sample (Kapler 1974; Matsuoka 1998b). This relationship may have been relevant for the rather slow surface velocities recorded at HSW and HNE, with low silt and clay contents (c.5%) despite their steep slopes. A non-linear relationship between slope angle and solifluction has also been revealed for subarctic mountains at landscape scale (Aalto & Luoto 2014; Hjort et al. 2014).

A further reason for the comparably low solifluction rates despite the steep slope angles at HSW and HNE could be that the steeper a slope is, the better it is commonly drained, thus influencing soil moisture conditions, which are relevant for solifluction. This slope-draining relationship has been observed by, for example, Stocker (1984) at the nearby Lenkenspitz site (Fig. 1A). Stocker (1984) also pointed out that gelifluction is a very important solifluction component on more gentle slopes. Furthermore, higher surface velocities can also be expected at fine debris layers that lack an openwork clast layer at the surface (Matsuoka 1998b) and unrelated to slope angle. As shown in Fig. 3, with the exception of the completely vegetation covered site FAB, all sites had both coarse and fine debris material at the surface.

### Significance of the subsurface velocity profiles

At four of the five solifluction monitoring sites, the subsurface velocity profiles indicated downslope concavity. Particularly for site SES, the highest solifluction monitoring site (2595 m a.s.l.), the downslope concavity was very pronounced. There, surface velocities measured at the tops of Rudberg columns (V_S (tube)_) were substantially lower than surface velocities measured by painted stones. This result was related to the lack of a closed vegetation cover, meaning that needle-ice creep was more efficient (Matsuoka 2001). A high relevance of needle-ice creep for solifluction at site SES was also indicated by the rather high numbers of FTCs and eFTCs. Downslope concavity is typical for slopes with little vegetation, where soil freezing and thawing is from the surface downwards (Harris et al. 2003).

Downslope concavities, albeit less distinct, were revealed for the north-west exposed site ELF, the north-east exposed site HNE, and the south-west exposed site HSW. At these three sites the vegetation cover was not entirely closed and therefore the surface was potentially prone to needle-ice creep (Figs. 3 and 4). Additionally, the relevance of needle-ice creep for HSW and HNE was indicated by the generally higher V_S (stone)_ rates compared with the V_S (tube)_ values (Fig. 9). The numbers of diurnal FTCs at ELF, HNE, and HSW were similar (Table 7).

The velocity profiles measured at FAB were substantially different from those measured at the other four sites, and lacked any indication of downslope concavity. Although FAB was not the lowest site in terms of elevation (Table 2), it was completely vegetated, thus preventing needle-ice creep, despite the fact that the highest numbers of FTCs and eFTCs were measured at this site. This observation showed that the ground surface conditions were more relevant than superficial freeze-thaw cycles.

### Possible controls for solifluction in the Hohe Tauern mountain range

A comparison of the results of the study with results from earlier studies by Stocker (1973; 1984) in the Lenkenspitz area, in the southern part of the Hohe Tauern range (Fig. 1A), revealed the following. Stocker measured the angle change of rods and tubes in south-west facing slopes and west-facing slopes consisting of mica schist weathering products. At a site with gelifluction (slope 25–29°), he measured mean annual rates of 5 cm yr^−1^. At a further four sites, all of which were steeper and drier than the first site (maximum 37°), frost creep dominated, with mean annual values increasing from 1.1 cm yr^−1^ at 1900 m a.s.l. to 1.8 cm yr^−1^ at 2100 m a.s.l. A maximum D_M_ of 60 cm was given, although the role of needle-ice creep was not discussed by Stocker (1973; 1984). According to Stocker (1984), the surface velocity at his sites depended on soil moisture, wind exposition and elevation. The V_S (tube)_ and D_M_ values from my study are generally in accordance with the gelifluction-influenced higher V_S_ values and the D_M_ given by Stocker (1984). In comparison with the values in the study reported here, the values given by Stocker (1984) suggest that both frost creep and gelifluction are relevant for the total surface velocity at the five studied solifluction lobes. The soil at ELF seemed to be relatively dry during the spring-to-summer melting season, as judged from the ZCP and STP data, and therefore gelifluction was possibly of minor importance. By contrast, data from SES, HSW, and HNE revealed longer durations of the ZCP and the STP, suggesting more moist conditions within the soils. However, the steeper slopes at HSW and HNE might have drained faster and led to a reduction in gelifluction rates.

Veit et al. (1995) pointed out that solifluction at the monitoring site Glorer Hütte c.2640 m a.s.l. (Fig. 1B), located 5.5 km north-west of HSW and HNE, and 5 km south-west of FAB, is dominated by gelifluction with a maximum D_M_ of 90 cm (mean 40–60 cm) and annual surface velocities of 2–80 cm yr^−1^. The main displacement of their studied solifluction landforms (consisting of micaschist and phyllite) takes place within some days to weeks during the early summer thawing period, although frost heave occurs during initial freezing in early winter and during meltwater infiltration into the frozen ground in spring (Jaesche et al. 2003). Veit et al. (1995) and Jaesche et al. (2003) note further that the later the winter snow cover starts to form, the higher the V_S_ rate in the subsequent spring. This relates to the fact that snow-free and/or snow-poor conditions in autumn and/or early winter favour deeper ground cooling and ground ice formation. Furthermore, Veit et al. (1995) revealed that the cooler the temperature in June and July, the higher the solifluction rate related to slower thawing of the ground. This potential relationship was also tested in the study reported here.

The observations made at the Glorer Hütte site are partly confirmed by the relationships presented in Fig. 8H (positive correlation of surface velocity and length of zero curtain period in spring), Fig. 8I (positive correlation of surface velocity and length of seasonal thawing period), Fig. 8J (positive correlation of surface velocity and beginning of the winter snow cover), and Fig. 8L (negative correlation between volumetric velocity and mean ground temperature in early summer). The latter relationship might be seen as a proxy for slow warming, implying that the frozen soil needs a longer time span to thaw after snowmelt (Jaesche et al. 2003). By contrast, other relationships revealed in the study and presented in Fig. 8 do not necessarily support the earlier findings at the Glorer Hütte site.

Lateral influx of meltwater (e.g. from a snow patch further upslope) might be essential for gelifluction (Jaesche et al. 2003; Matsuoka et al. 2005). Such potential influx of water was not considered in the study, due to missing snow data. Based on numerous field visits to the five sites in July and August each year since 2006, it can be at least stated that there were no perennial snow patches near the five solifluction sites, and all the winter snow near the five solifluction lobes melted by August. This conclusion also supports the results of automatic optical monitoring of the seasonal snow cover in the Hinteres Langtal cirque (Kellerer-Pirklbauer & Rieckh 2016). Therefore, a warm summer in terms of a hot August, which in most cases was the warmest month (i.e. MGST-WM), a warm autumn, or a high number of thawing degree days are all not necessarily good parameters for solifluction analyses because the warmest part of the year is of little relevance for solifluction at the studied landforms. This has been shown by, for example, Jaesche et al. (2003), who pointed out that solifluction movement at their Glorer Hütte site was primarily related to high soil moisture content, which itself was related to adequate ground freezing and ice lens formation during early winter, thus preparing the soil for displacement.

### Correlating solifluction rates with ground temperatures: strengths and weaknesses

The results of the correlation analysis clearly showed that four years of data (including several data gaps related to technical problems common in such environments (cf. Jaesche et al. 2003)) were not enough to allow for a statistically significant assessment of the influence of ground temperature derived parameters on solifluction rates. Site SES was by far the highest site (2595 m a.s.l.) of the five solifluction monitoring sites and had the greatest possible freezing depths (DF mean of 316 cm) and located in a marginal permafrost setting as indicated by the MAAT values. There, warmer years in terms of higher number of TDDs (Fig. 8B) or less cool autumns (lower MAGT-O) may have caused the formation of deeper active layers and greater depths of movement. This hypothesis is supported by the results of research conducted by Ridefelt et al. (2009), who revealed a positive correlation between surface velocity and MAAT and between surface velocity and autumn air temperature at a study site in northern Sweden.

In the case of site HSW, there seems to have been a positive correlation between warmer ground temperature conditions (in terms of fewer FDDs, higher MGST-WM, and higher MGST-CM), longer zero curtain periods (ZCPs), and longer seasonal thawing periods (STPs) and higher solifluction rates at the surface. By contrast, a negative correlation was revealed for the volumetric velocity and the early summer ground temperature. HSW was generally a warm site characterized by a minor content of clay and silt. The relationships shown in Fig. 8E-L point to the main drivers as the duration of the ZCP, the duration of the STP, the date of the SOD, the number of FTCs, and the temperature during the early summer thawing season (MGST-JJ), all of which influenced to some extent the slope moisture conditions and hence the solifluction rate. These explanations are in agreement with the results from the Glorer Hütte site, where the main driver for solifluction is the spring melting period (Veit & Höfner 1993; Jaesche et al. 2003).

The possible explanations for the seeming relationships between solifluction rates and ground temperature based parameters were only weakly supported by the data, which thus indicates that a four-years data series is short and that substantially longer data series as well as additional measurement techniques (shown by, for example, Stingl et al. 2010; Matsuoka 2011) are essential for a more in-depth analysis of environmental drivers of solifluction. However, the results presented here do not necessarily conflict with earlier results from the region and even support earlier findings with similar short time series. In addition, it should be noted that longer data series do not necessarily deliver clearer relationships between solifluction and temperature data (Ridefelt et al. 2009).

### Comparison with earlier studies at a global scale

**Fig. 10.**
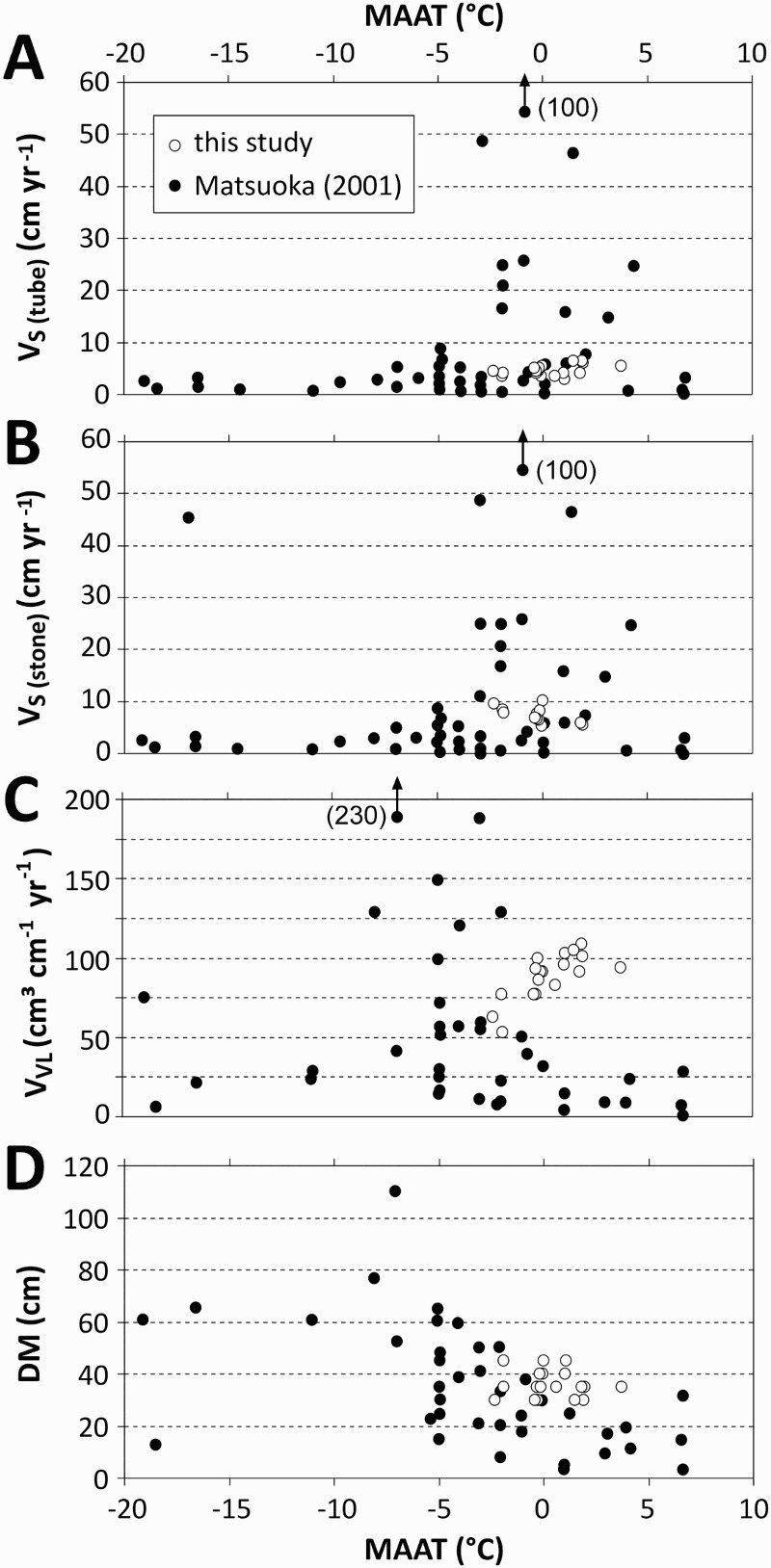
Solifluction as a function of mean annual air temperature (MAAT) as revealed in the study in comparison with a global data set (based on 48 references) compiled by Matsuoka (2001); V_S (tube)_ = surface velocity based on Rudberg columns or flexible tubes; V_S (stone)_ = surface velocity based on painted lines; V_VL_ = volumetric velocity; D_M_ = maximum depth of movement

**Fig. 11.**
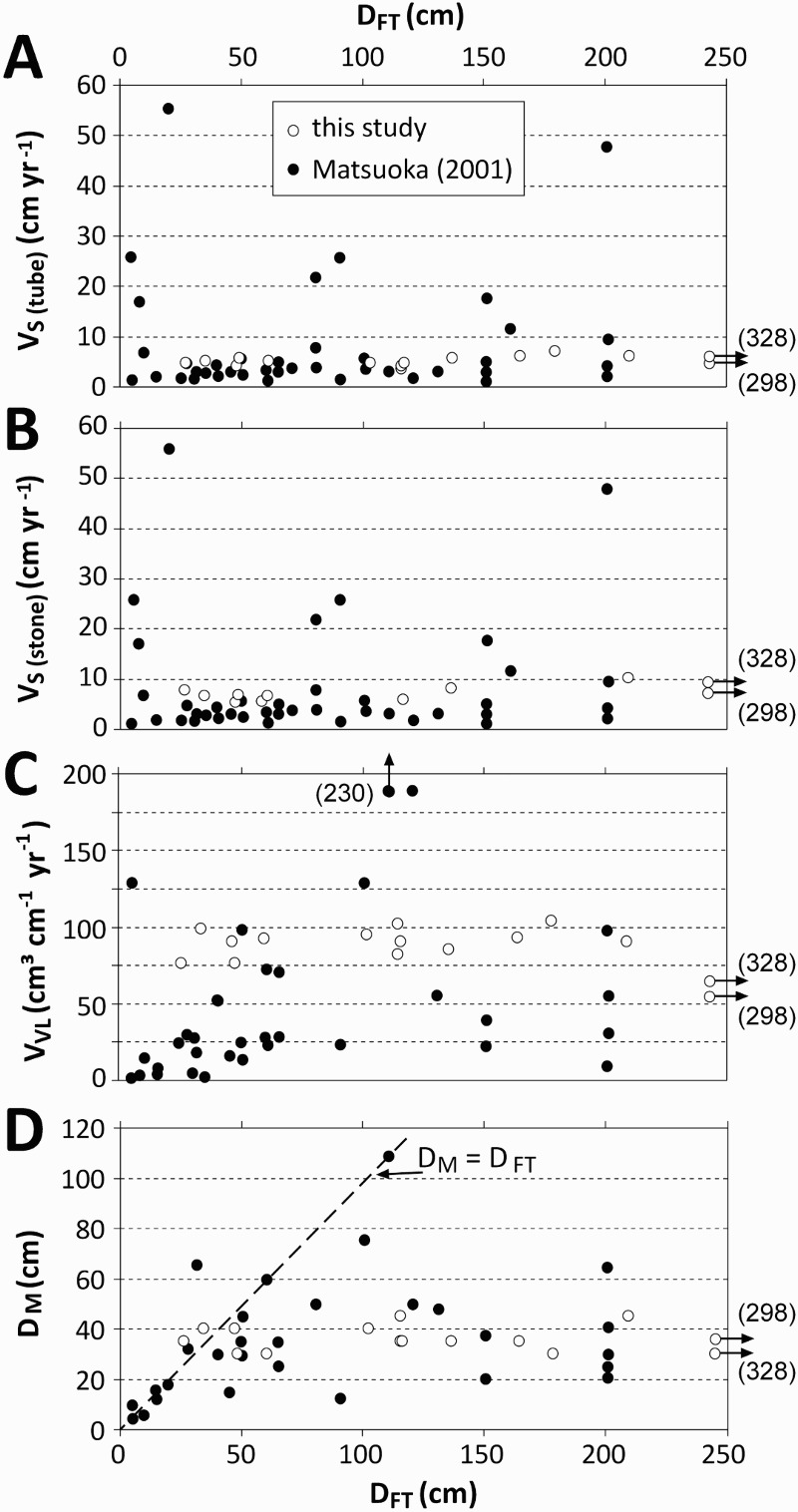
Solifluction as a function of depth of thawing (D_T_) or freezing (D_F_) as quantified in the study in comparison with a global data set (based on 48 references) compiled by Matsuoka (2001); V_S (tube)_ = surface velocity based on Rudberg columns or flexible tubes; V_S (stone)_ = surface velocity based on painted lines; V_VL_ = volumetric velocity; D_M_ = maximum depth of movement

To see the solifluction measurement results of the study in a global perspective, the data results were combined with data from a global dataset compiled by Matsuoka (2001). His dataset is based on 48 references, of which 23 are relevant for polar and subpolar solifluction data and 25 references have data from mid-latitude to tropical mountains. Matsuoka (2001) discussed the relationship between solifluction and the MAAT as well as the depth of thawing (D_F_) or freezing (D_T_). Figures 10 and 11 show the data compiled by Matsuoka (2001) in combination with the data from the study reported here. In his dataset, Matsuoka (2001) did not distinguish between V_S (stone)_ and V_S (tube)_, and therefore his data shown in Fig. 10A and 10B and Fig. 11A and 11B are identical. The comparison shows that the surface velocities revealed by the study reported here may be regarded as typical in the respective MAAT range. By contrast, the observed D_M_ values were slightly higher than those from earlier studies with higher MAAT values. The volumetric velocities quantified in the study reported here were substantially higher at positive MAAT values compared with earlier studies, as shown in Fig. 10C. This finding may be related to the fact that in both cases – the study and the global review by Matsuoka (2001) – the air temperature was not always measured directly at the solifluction sites but was only interpolated from data from neighbouring stations, using an assumed lapse rate of 6 °C km^−1^. A simple change in the lapse rate would have influenced the distribution pattern. Matsuoka (2001) pointed out that the air temperature data were not always cited in the literature he consulted. Therefore, he estimated the MAAT from meteorological data at nearby weather stations using the above-mentioned lapse rate. However, no information was given by Matsuoka (2001) about the precise data source and whether 30-year climate normals or individual years had been used in his MAAT compilation. Matsuoka (2001) quantified this uncertainty and mentioned an estimated error not greater than ±2 °C. Harris et al. (2008) revealed that at their studied landform the surface velocity, volumetric velocity, and maximum depth of movement increased substantially from the rear of a studied lobe to the frontal zone, although the MAAT was basically identical.

Considering the above-described data restrictions and uncertainties, it is apparent that the results from the study could fit better in the global compilation. Furthermore, this shows that it is challenging to compare MAAT data that are extrapolated from nearby automatic weather stations in a potentially different topographic setting (e.g. a summit station versus slope site) with MAAT data measured directly at a solifluction monitoring site. Therefore, in a strict sense, it is only meaningful to correlate at most (cf. Harris et al. 2008) air temperature data and solifluction data only from the same site. My observations in the study as well as the strong seasonality of solifluction rates in the study region (as discussed above) also indicate that the MAAT is not an adequate indicator of solifluction-climate relationships, although some statistically significant correlations have been revealed (e.g. Ridefelt et al. 2009).

The combination of solifluction data with DF data based on on-site data should therefore be substantially more reliable. The comparison of solifluction rates and the depth of thawing (D_F_) or freezing (D_T_) from the study with the data from Matsuoka (2001) reveals inconspicuous results for surface velocity and depth of movement. Additionally, the relationships found between the volumetric velocity and D_F_ and/or D_T_ in my study are more or less in accordance with findings from earlier studies. However, it is not always easy to detect how the depth of thawing or freezing was quantified in earlier studies, thus introducing further uncertainty. From Matsuoka's Figure 5 (2001, 118), it seems that the depth of freezing or thawing had been estimated for depths ≥ 150 cm because the data points were accumulated around the 150 cm and 200 cm values. This also highlights the problem of making comparisons between global datasets or datasets from two studies if complete background data (e.g. detailed description of the methodological approach) are missing.

## Conclusions

I have drawn the following conclusions from the study.

Non-electronic methods for solifluction monitoring at five different solifluction lobes in seasonal frost (four sites) and warm permafrost (one site) environments were successfully applied during a four-year monitoring period. Despite the limitation of the low temporal resolution of the chosen approaches (i.e. annual), the solifluction rates at the five sites did not correlate with each other and hence did not depend on regional climatic controls but on site-specific controls.

The near-surface downslope concavity of the velocity profiles was governed primarily by the vegetation cover and was not related to elevation, aspect, or the number of freeze-thaw cycles.

The mean surface velocities ranged from 3.5–6.1 cm yr^−1^ measured by the tops of flexible tubes or Rudberg columns to 6.2–8.9 yr^−1^ measured by painted lines. The values derived from the painted lines were 1.5–2.0 times higher at the same sites, suggesting a high relevance of needle-ice creep for those sites. The mean depth of movement was 32.5–40 cm, which indicated the high relevance of annual frost creep. Gelifluction, as a further solifluction component, was relevant at all five sites, although to a minor extent at the two steeper sites, HSW and HNE.

An almost linear relationship existed between surface velocity and slope angle on more gentle (≤ 21°) slopes. However, two sites with steeper slope angles (HSW – 32° and HNE – 34°) showed substantially smaller surface velocities, which were related to minor silt and/or clay contents and possible drier soil conditions hampering gelifluction.

The site with the lowest volumetric velocities (SES) was characterized by the highest interannual variability and located at or near warm permafrost. By contrast, the mass transport rates by solifluction in a seasonal frost environment at a lower elevation (ELF) were not only the highest ones at all five studied sites but also the most stable ones regarding interannual variability. This implies that sites with warm permafrost are not necessarily the sites with the highest mass transport.

Correlation analyses of solifluction data with different ground temperature derived parameters revealed that only few of the used parameters seem useful as solifluction-intensity parameters related to the seasonality of the solifluction. The mean annual air temperature, the sum of thawing and freezing degree days per year, and the mean ground surface temperature of the coldest and warmest months are of little use for such analyses. By contrast, the number of freeze-thaw cycles and effective freeze-thaw cycles, the depth of frost or thaw penetration, the length of the zero curtain period and of the seasonal thawing period in the soil in spring and early summer, the snow onset date in autumn, and the mean ground surface temperature in October all seem useful for such analyses.

The comparison with a global data set revealed that the surface velocities found in the study could be considered typical in the respective MAAT ranges. The observed depth of movement and the volumetric velocities were higher than those from earlier studies, particularly those conducted in warm climates. In relation to estimated MAAT values and the frequent seasonality of solifluction, I conclude that MAAT is not an adequate indicator of solifluction rate estimates. This implies that the correlation of climate-derived parameters with solifluction rates is not straightforward.
